# Supplementary material for: Migration and Invasion Enhancer 1 Is an NF-ĸB-Inducing Gene Enhancing the Cell Proliferation and Invasion Ability of Human Prostate Carcinoma Cells In Vitro and In Vivo
Source: Cancers (Basel). 2019 Oct 2;11(10):1486. doi: 10.3390/cancers11101486 (PMC6826896; doi:10.3390/cancers11101486)
Supplement: Supplementary file 1 [file cancers-11-01486-s001.zip › cancers-600514-supplementary-revised/cancers-600514-supplementary.docx]

**Supplementary Materials**


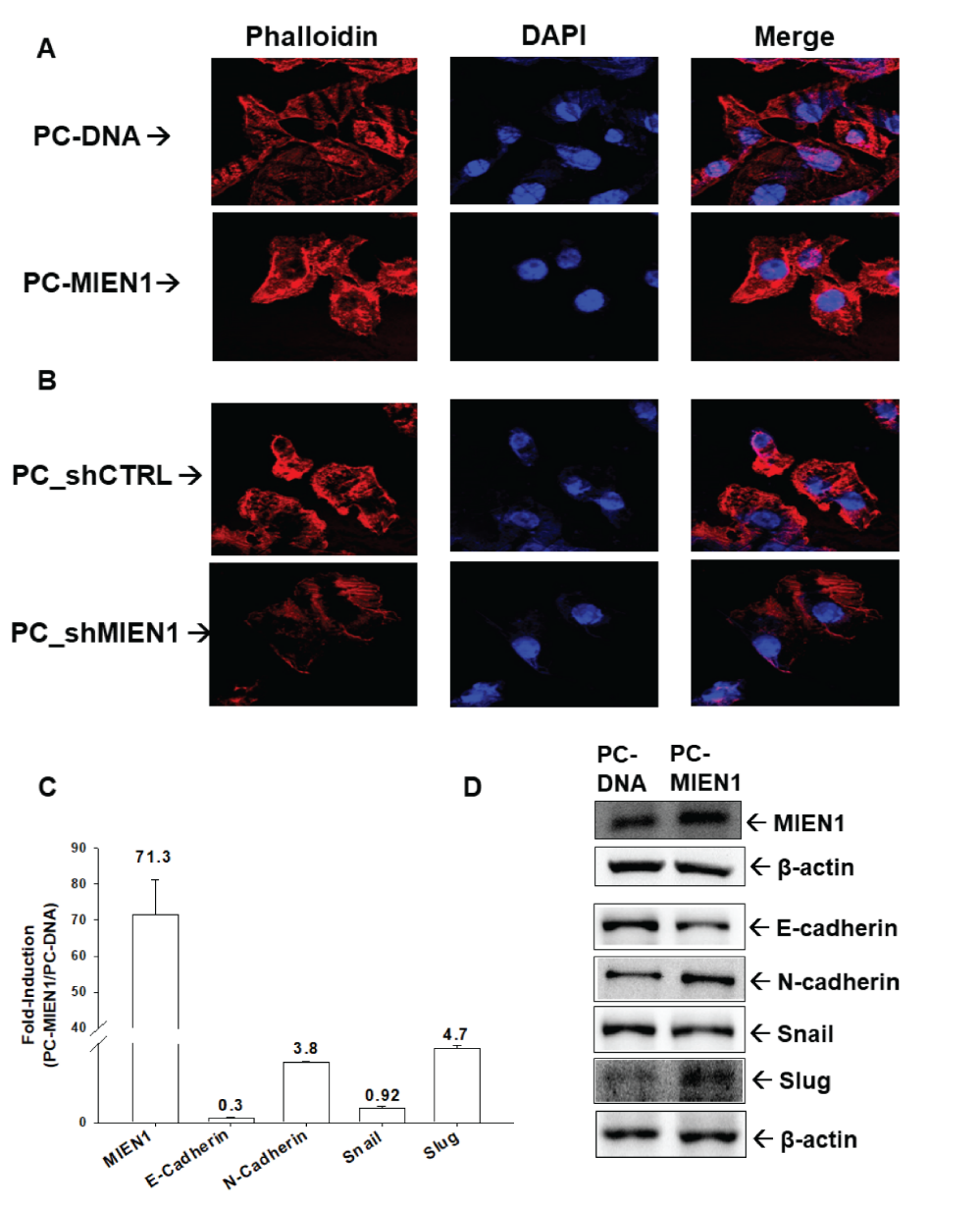


**Figure S1.** Effects of MIEN1 on actin cytoskeleton and epithelial–mesenchymal transition. The F-actin staining after MIEN1 was overexpressed (**A**) or knockdowned (**B**) in PC-3 cells. The expressions of MIEN1, E-cadherin, N-cadherin, snail, and slug in PC-DNA and PC-MIEN1 cells were determined by (**C**) RT-qPCR (± SE, n = 3; The number indicates the fold-induction) and (**D**) immunoblot assays.
