# Supplementary material for: Migration and Invasion Enhancer 1 Is an NF-ĸB-Inducing Gene Enhancing the Cell Proliferation and Invasion Ability of Human Prostate Carcinoma Cells In Vitro and In Vivo
Source: Cancers (Basel). 2019 Oct 2;11(10):1486. doi: 10.3390/cancers11101486 (PMC6826896; doi:10.3390/cancers11101486)

Figure 1A. PZ, CA, LN, PC, DU

MIEN1 (~15kD)

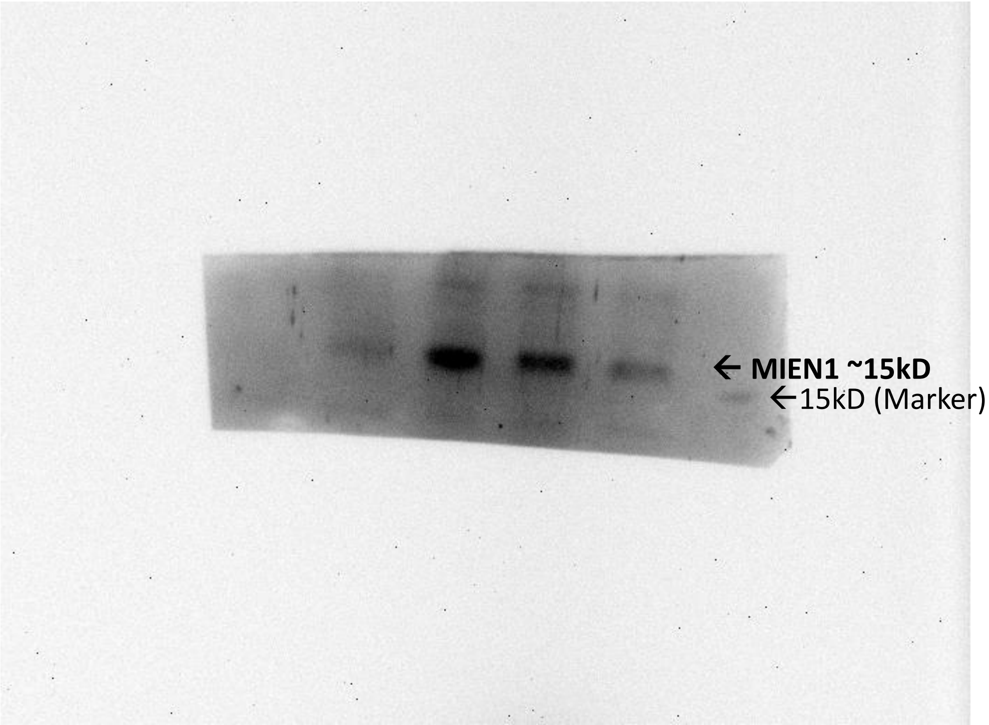

Actin (43kD)

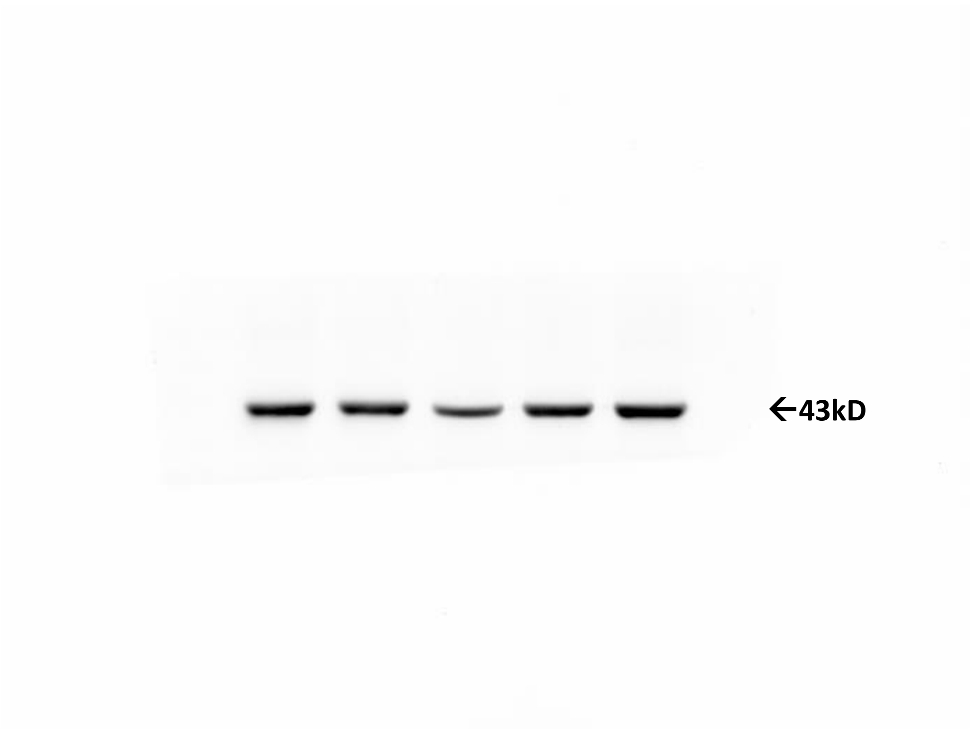

Figure 2C-1. PC-DNA, PC-IκBα

IκBα (41kD)

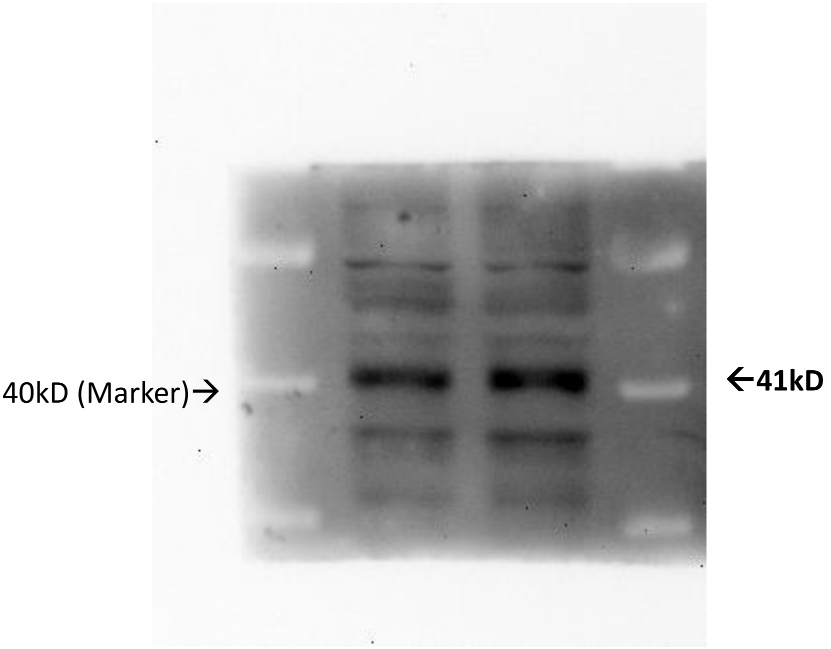

Actin (43kD)

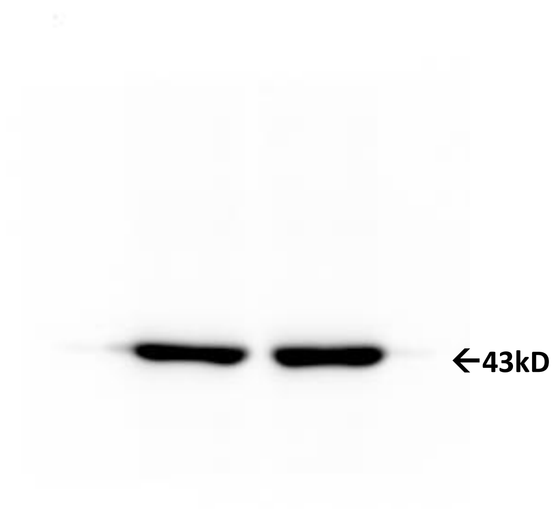

MIEN1 (~15kD)

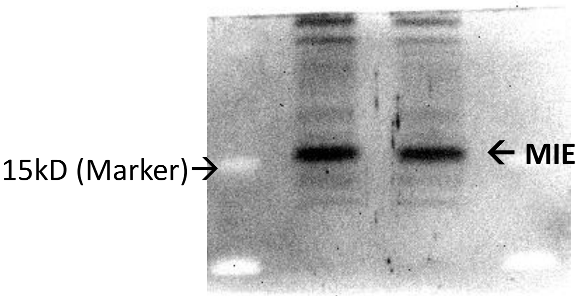

Actin (43kD)

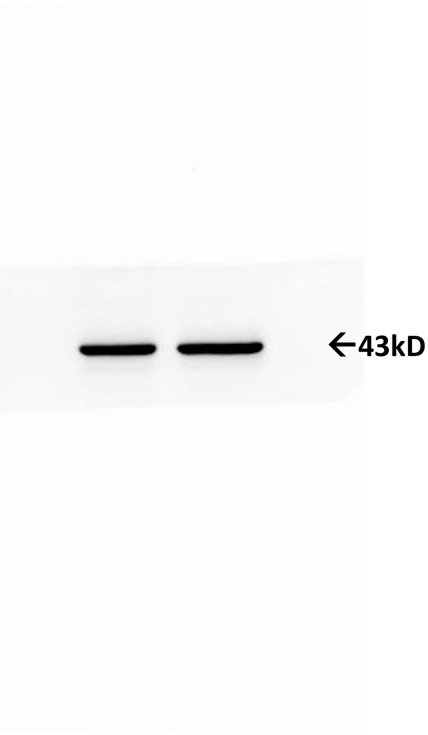

Figure 2C-2. PC-DNA, PC-NIK

NIK (125kD)

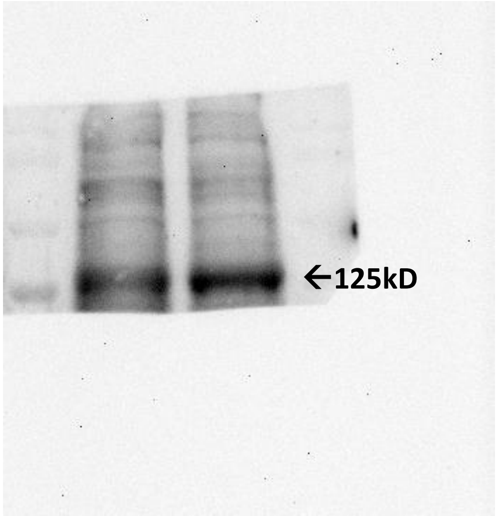

Actin (43kD)

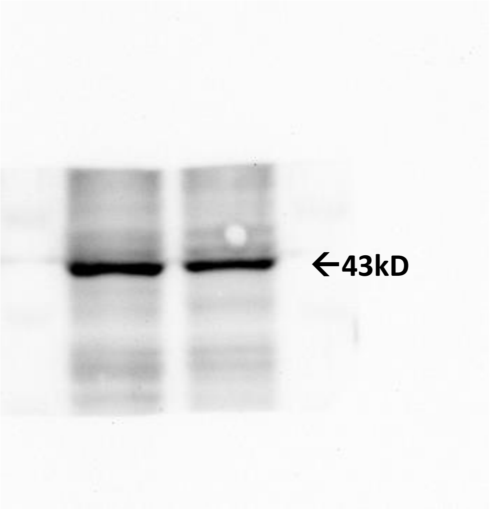

MIEN1 (~15kD)

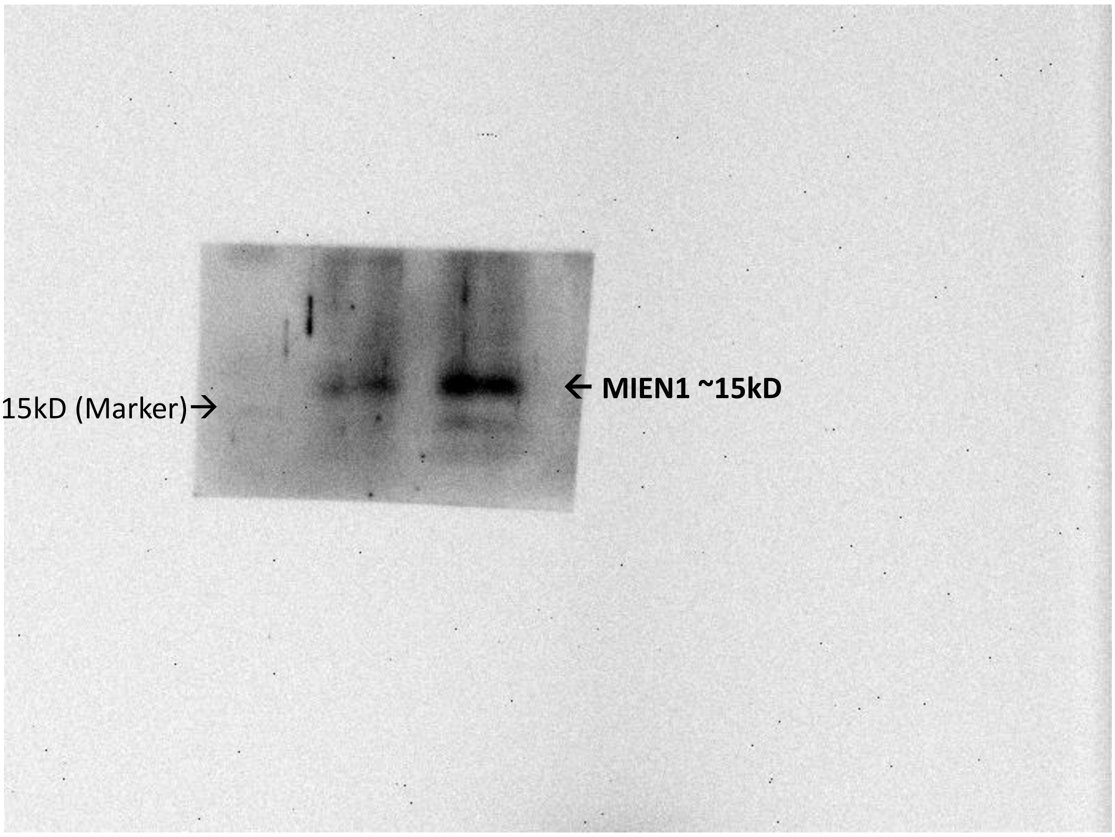

Actin (43kD)

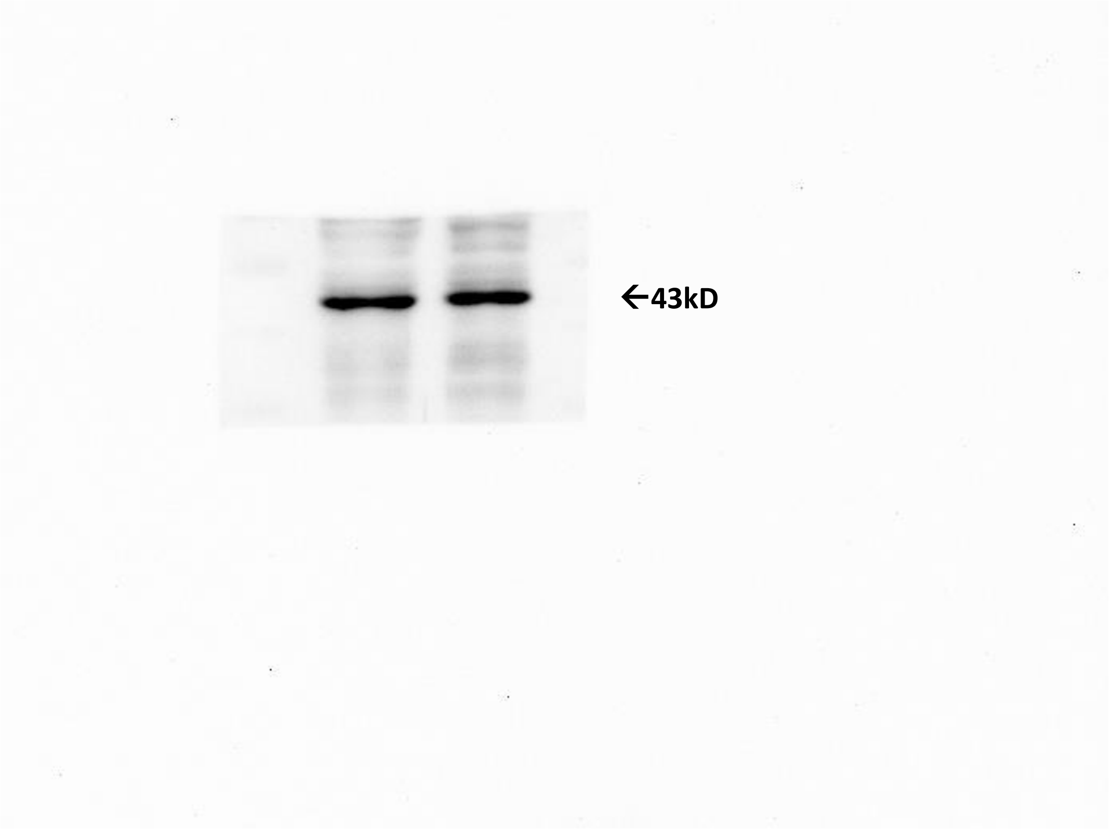

Figure 2E-1. LN-shCOL, LN-shMIEN1

MIEN1 (~15kD)

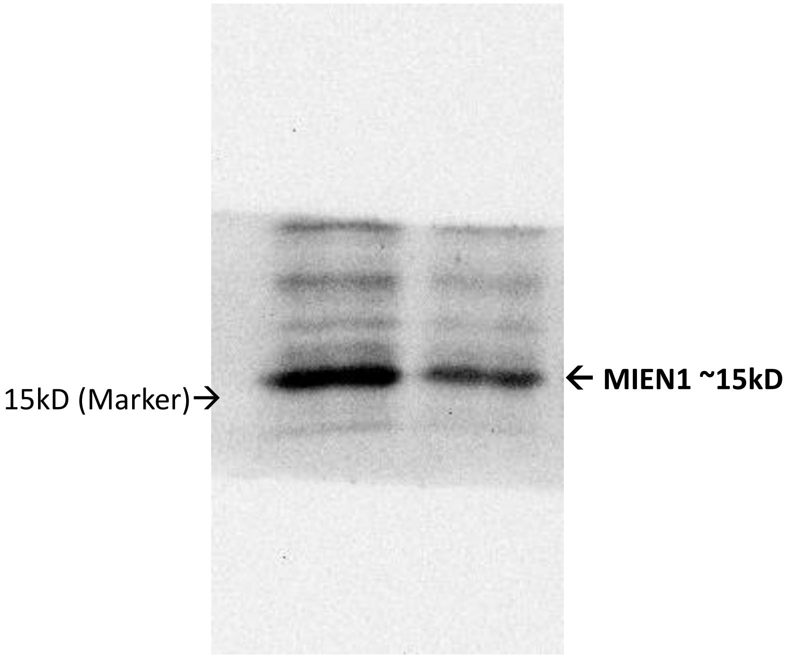

Actin (43kD)

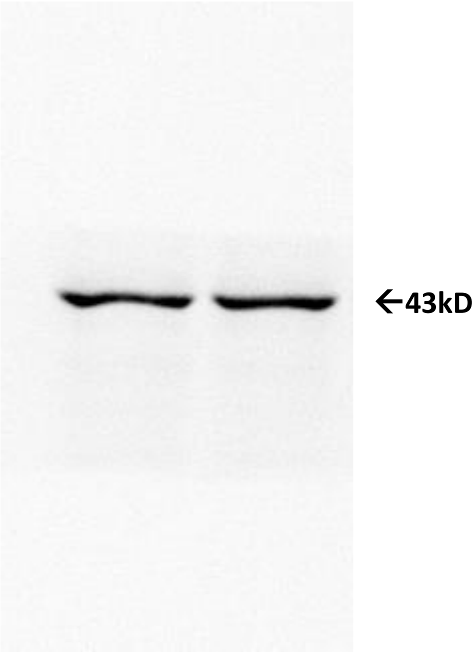

pAkt^{S473} (60kD)

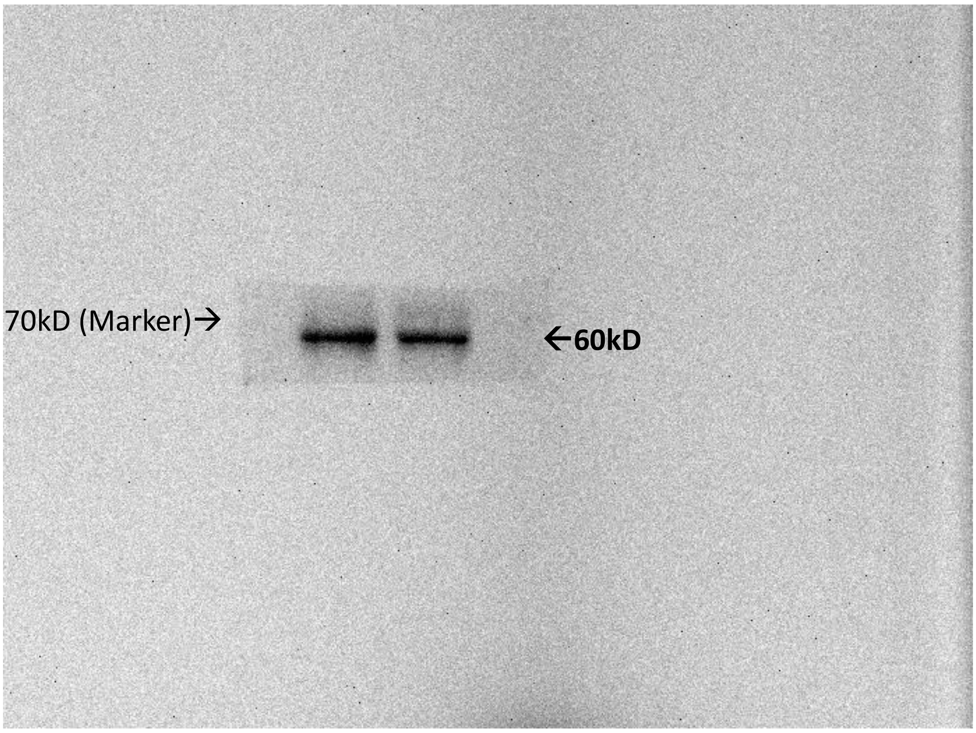

Akt (60kD)

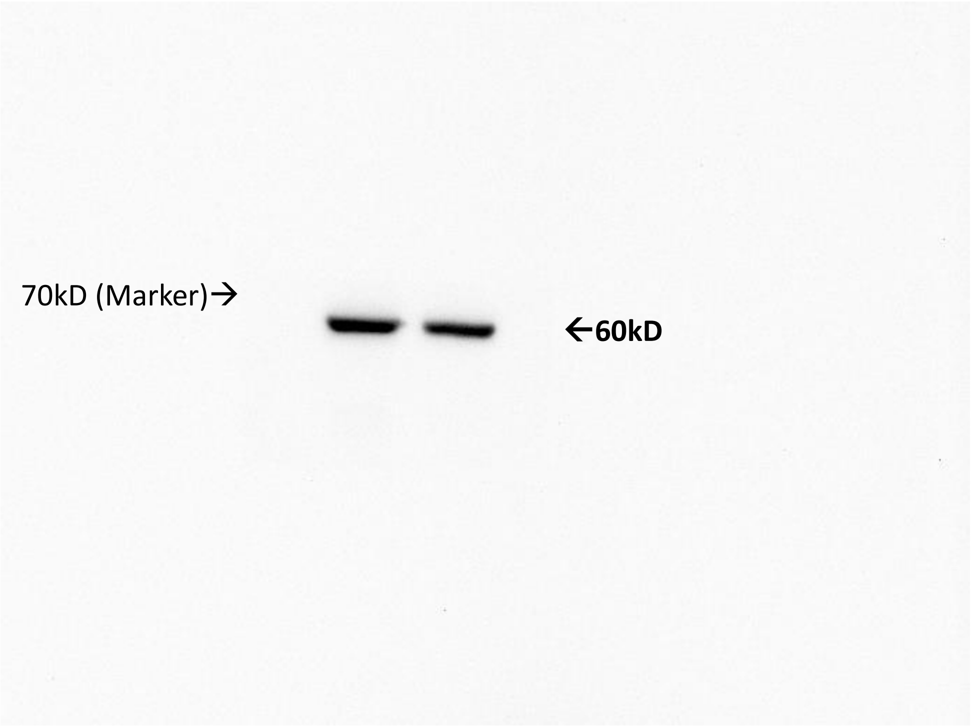

Figure 2E-2.   PC-DNA, PC-MIEN1

MIEN1 (~15kD)

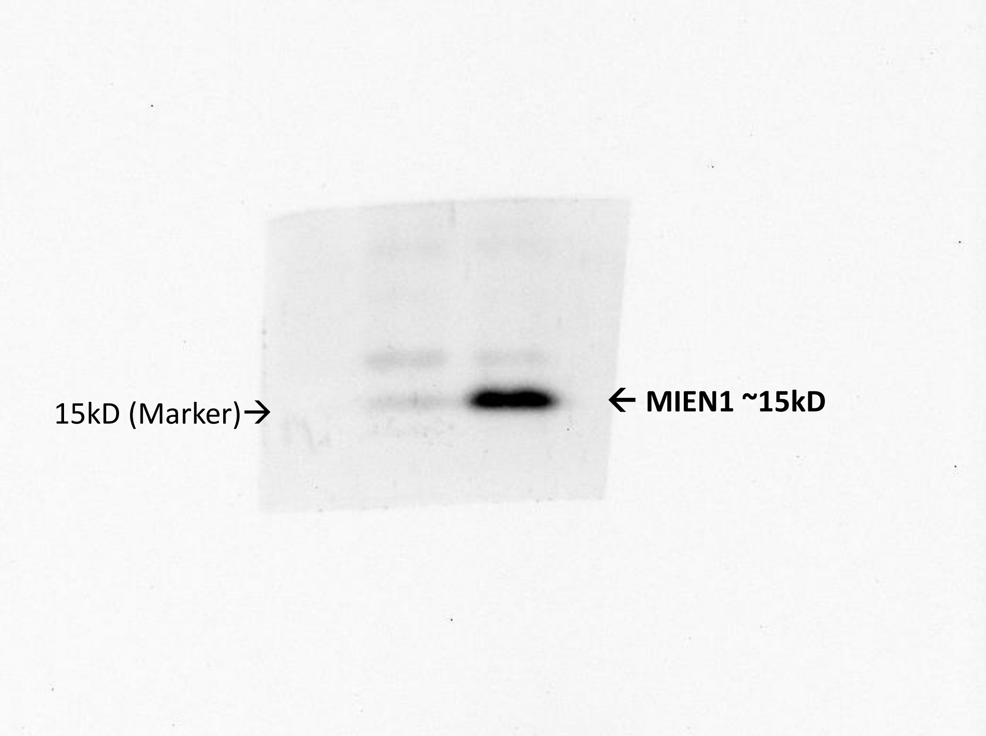

Actin (43kD)

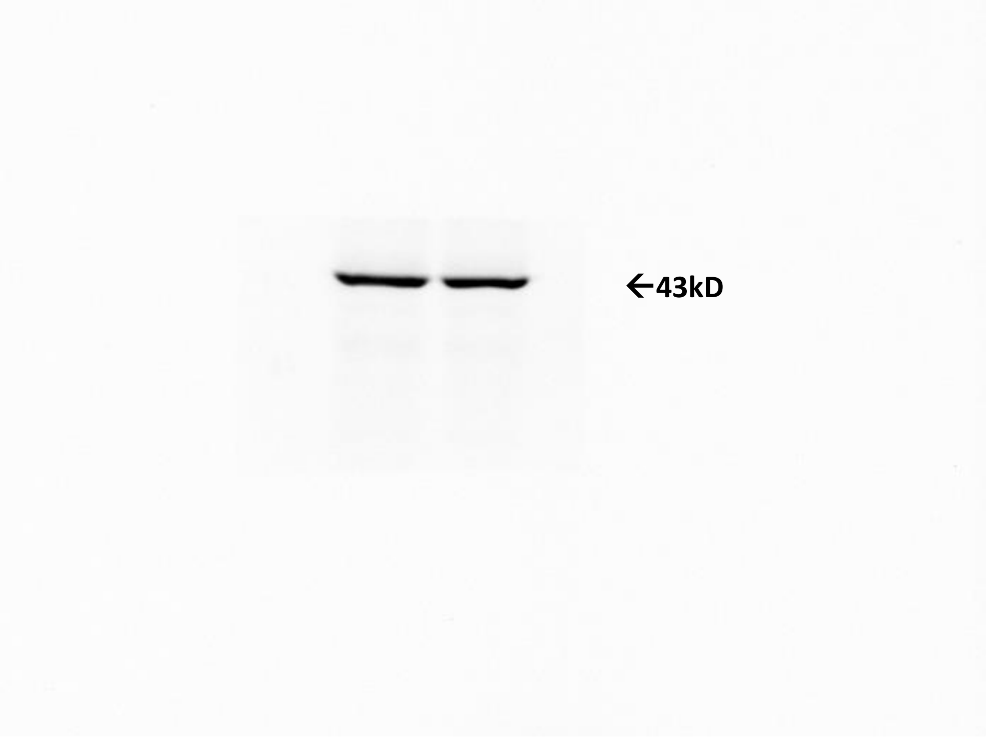

pAkt^{S473} (60kD)

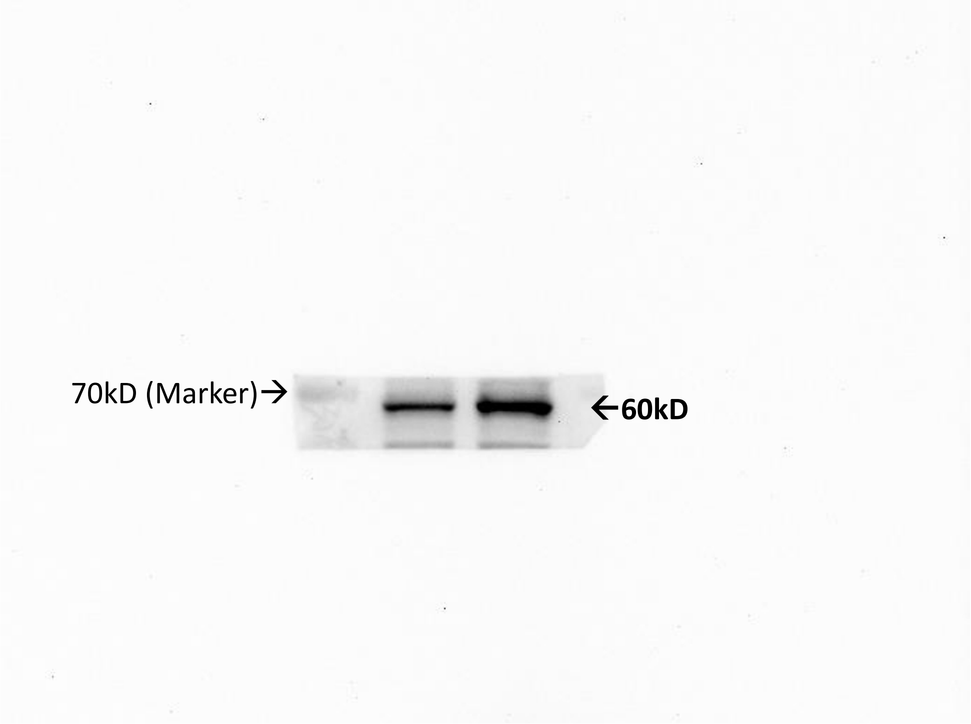

Akt (60kD)

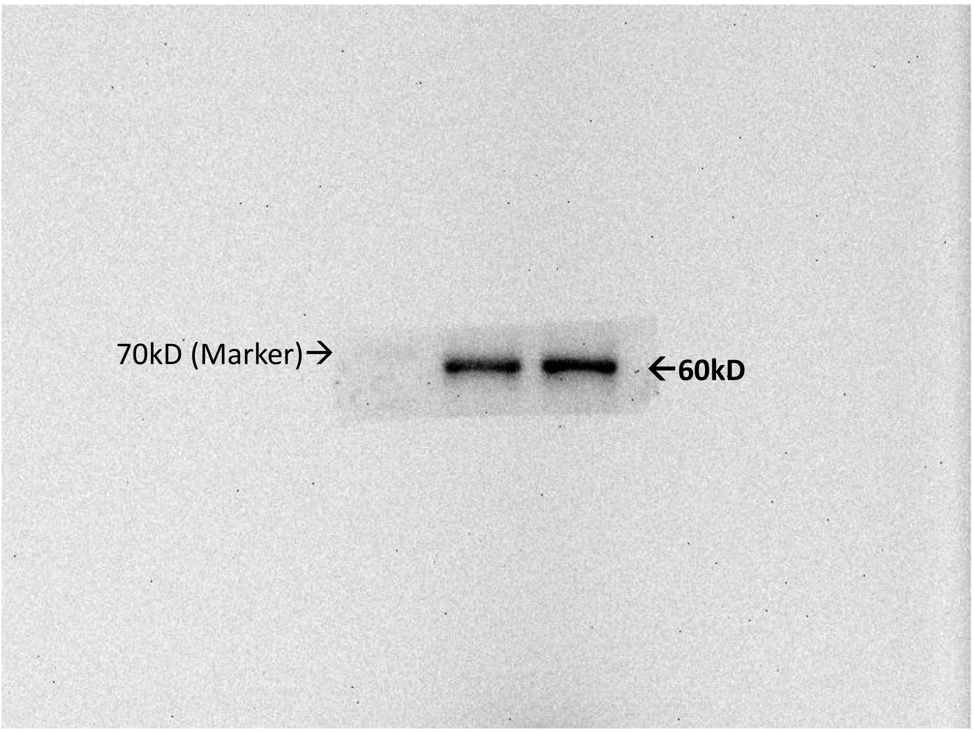

Figure 2E-3. PC-shCOL, PC-shMIEN1

MIEN1 (~15kD)

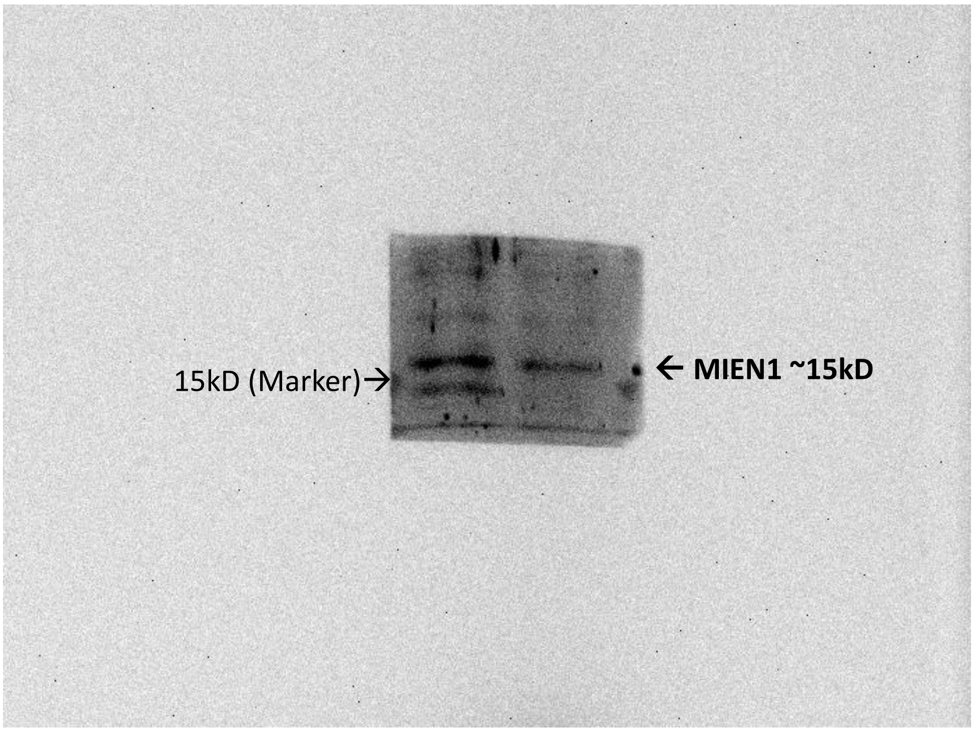

Actin (43kD)

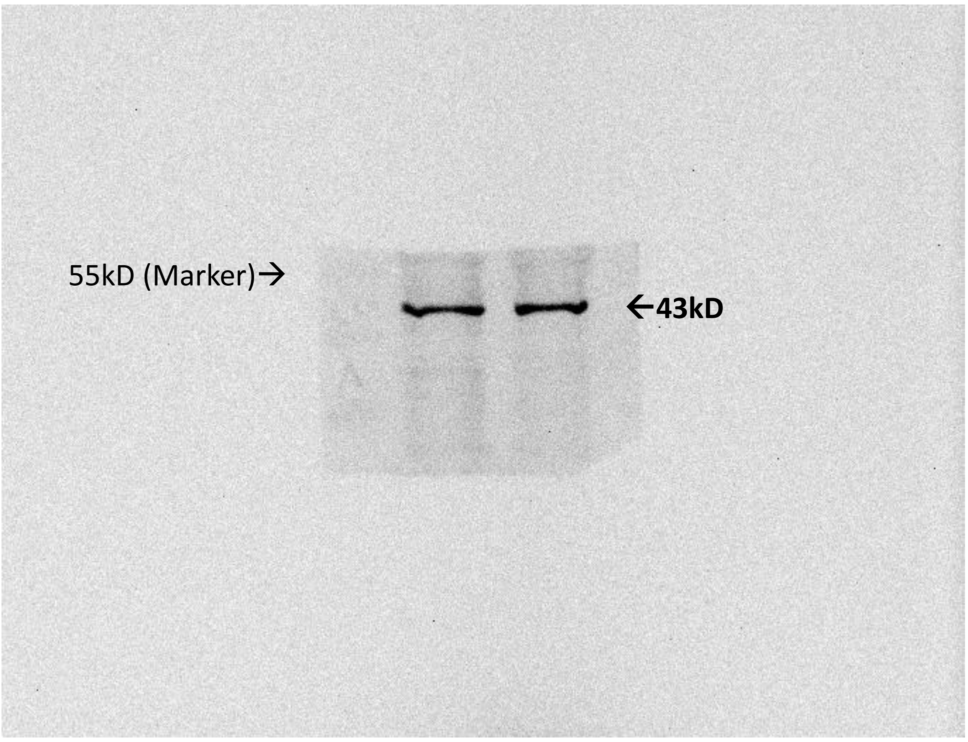

pAkt^{S473} (60kD)

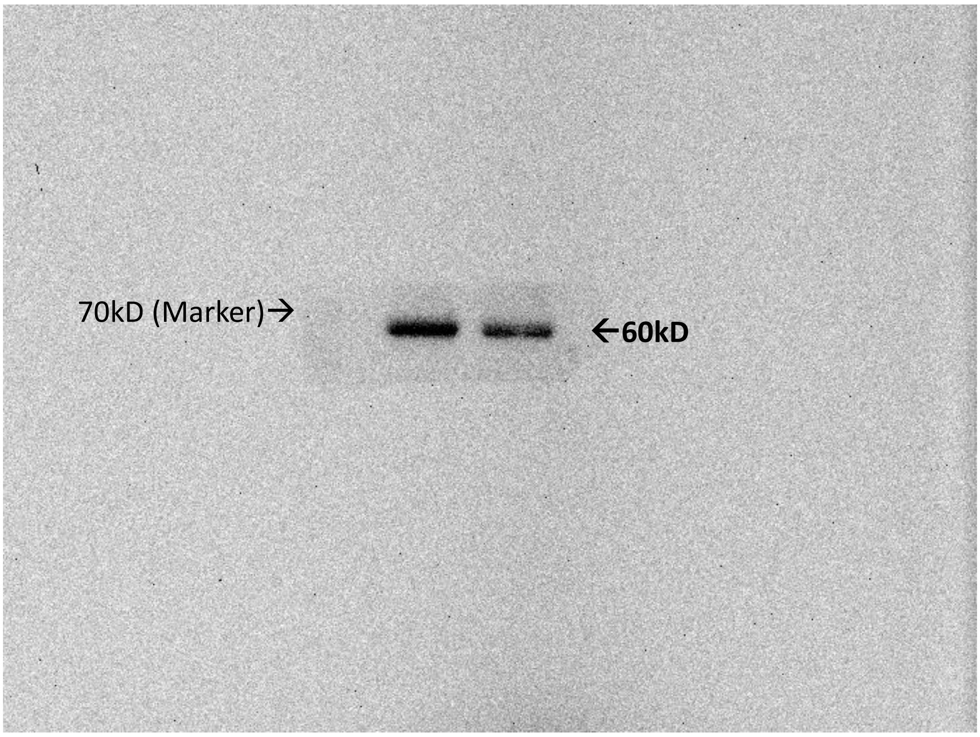

Akt (60kD)

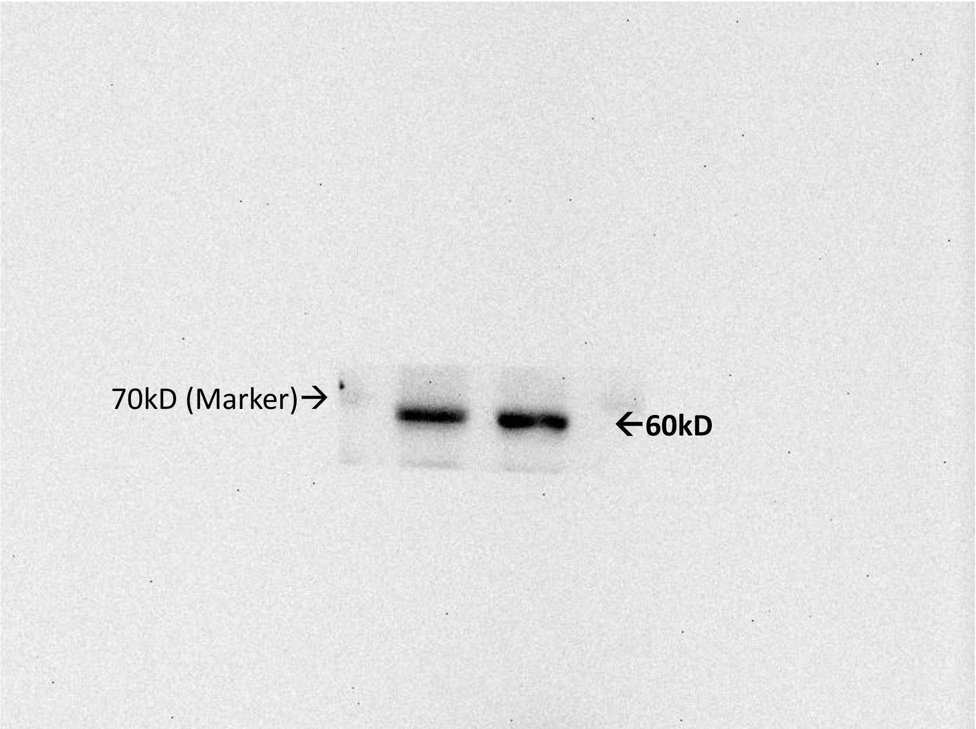

Figure 2F. PC-DNA, PC-MIEN1, PC-DNA+MK2206, PC-MIEN1+MK2206

pAkt^{S473} (60kD)

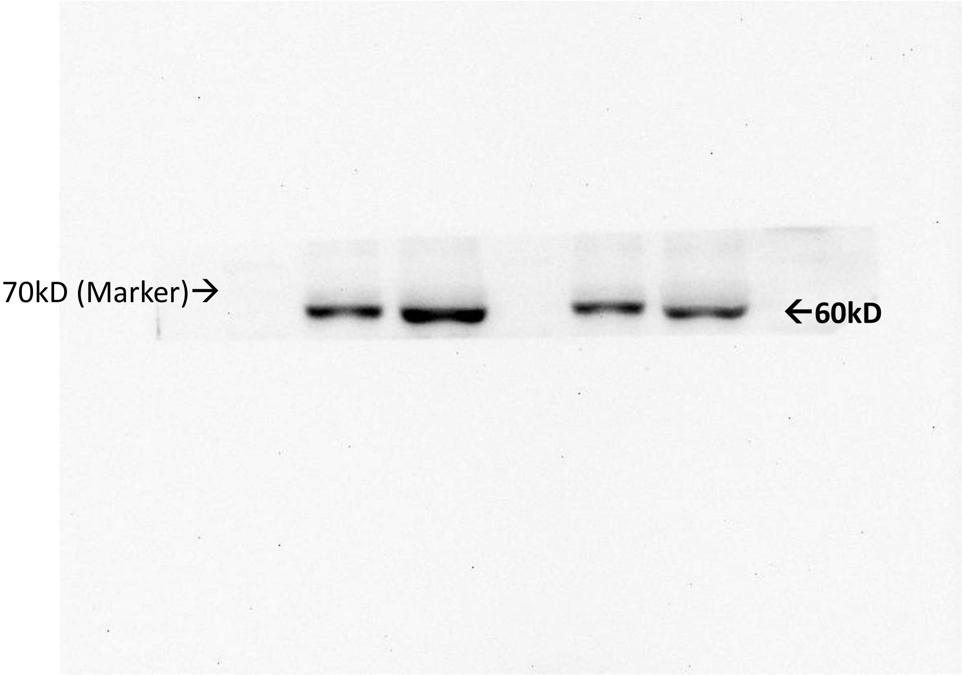

Akt (60kD)

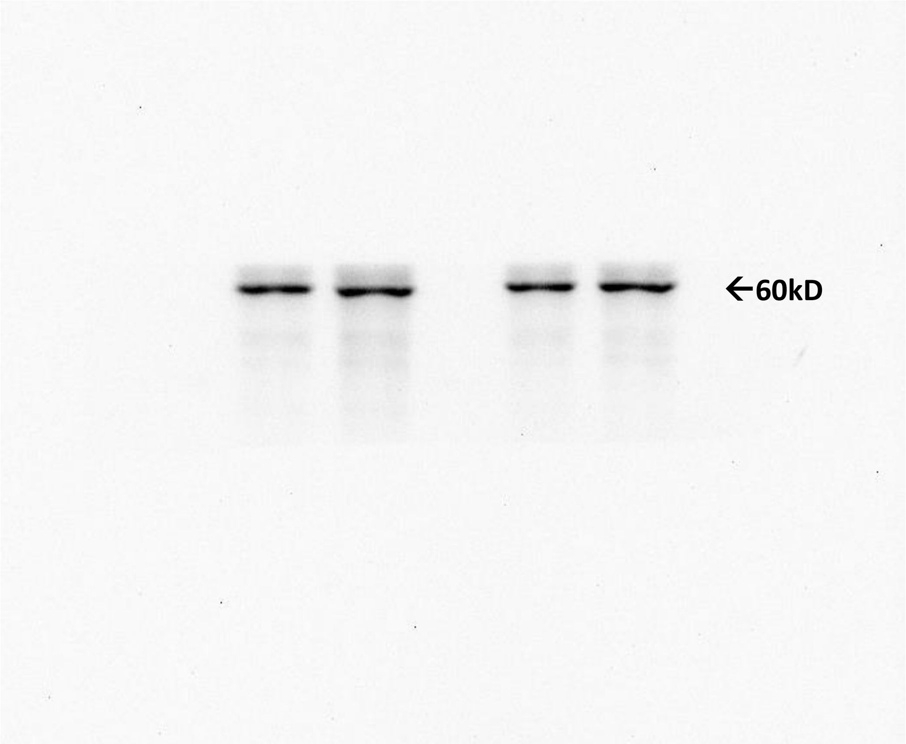

MMP9 (92kD)

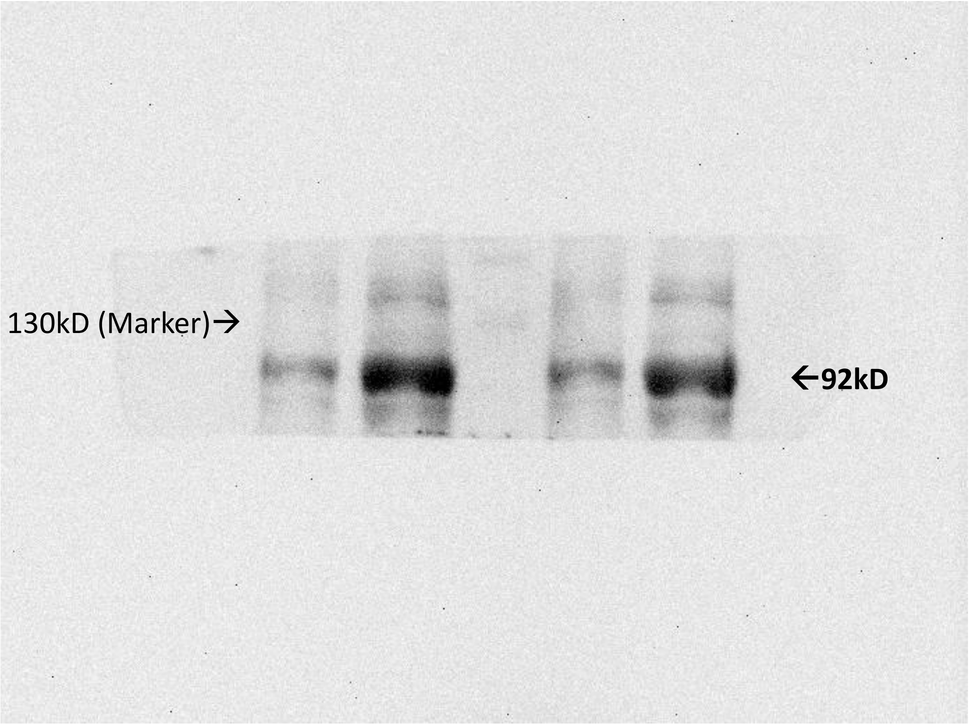

Actin (43kD)

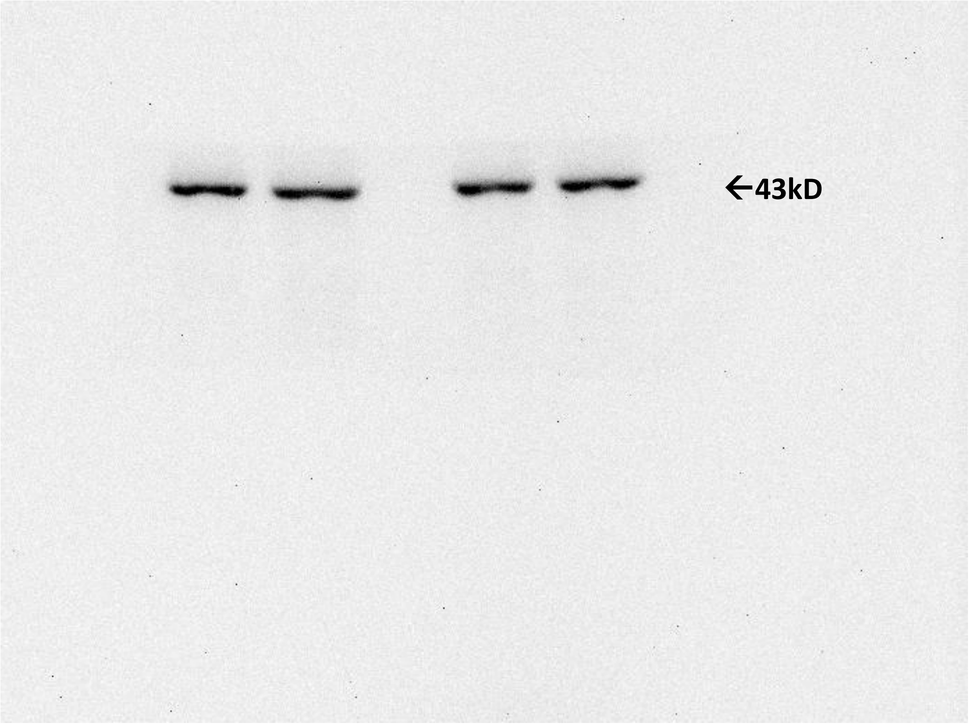

Figure 3A. LN-shCOL, LN-shMIEN1

MIEN1 (~15kD)

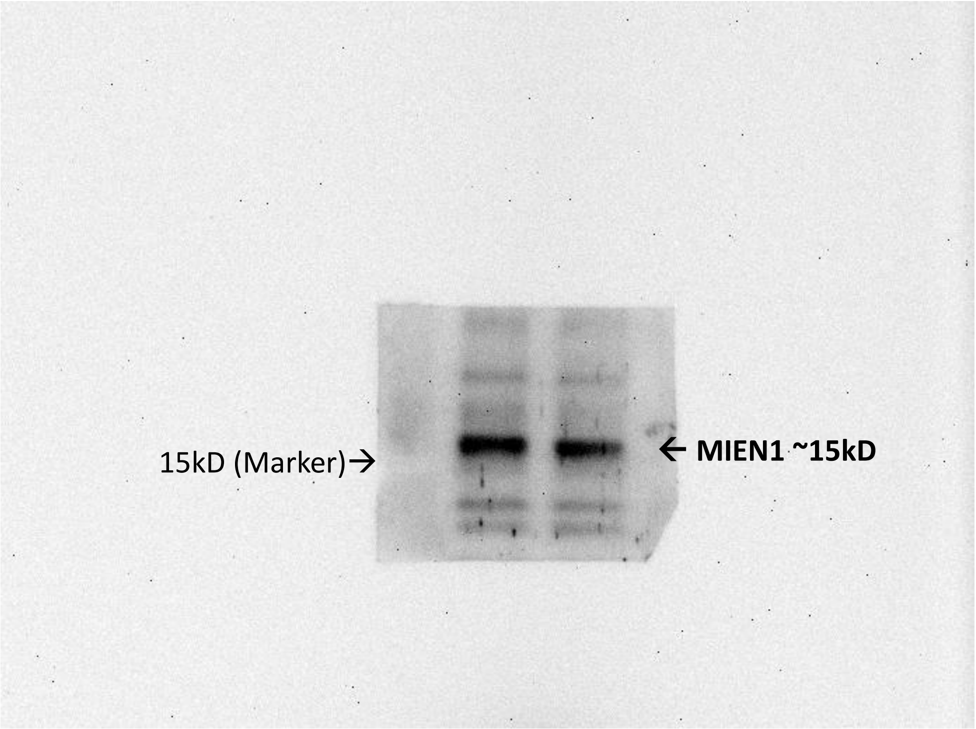

Actin (43kD)

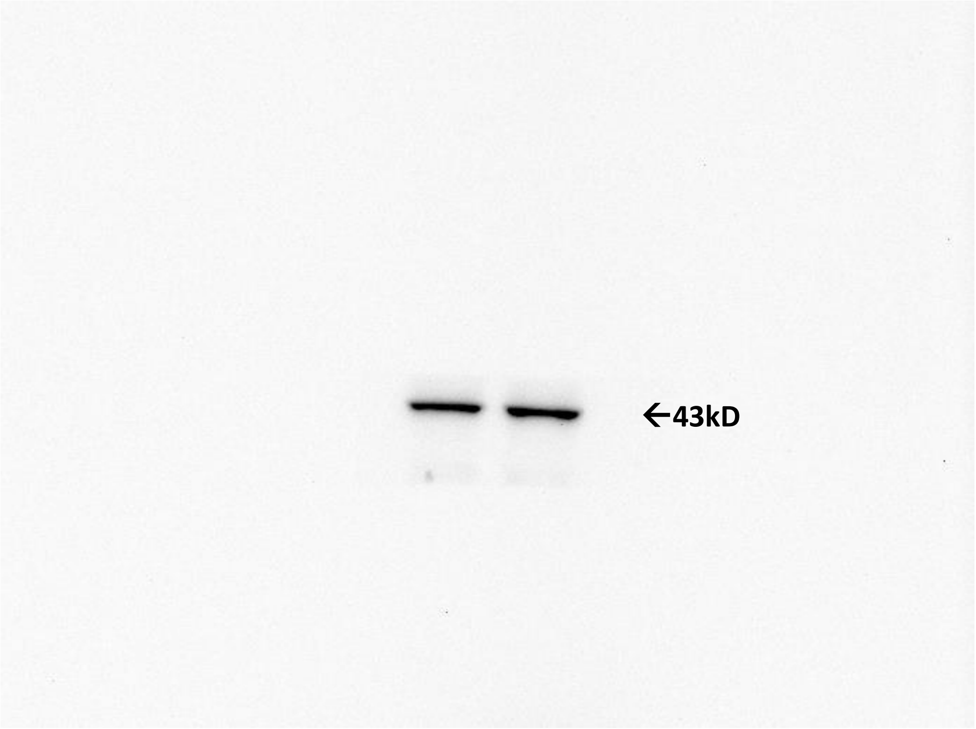

Figure 3B. PC-shCOL, PC-shMIEN1

MIEN1 (~15kD)

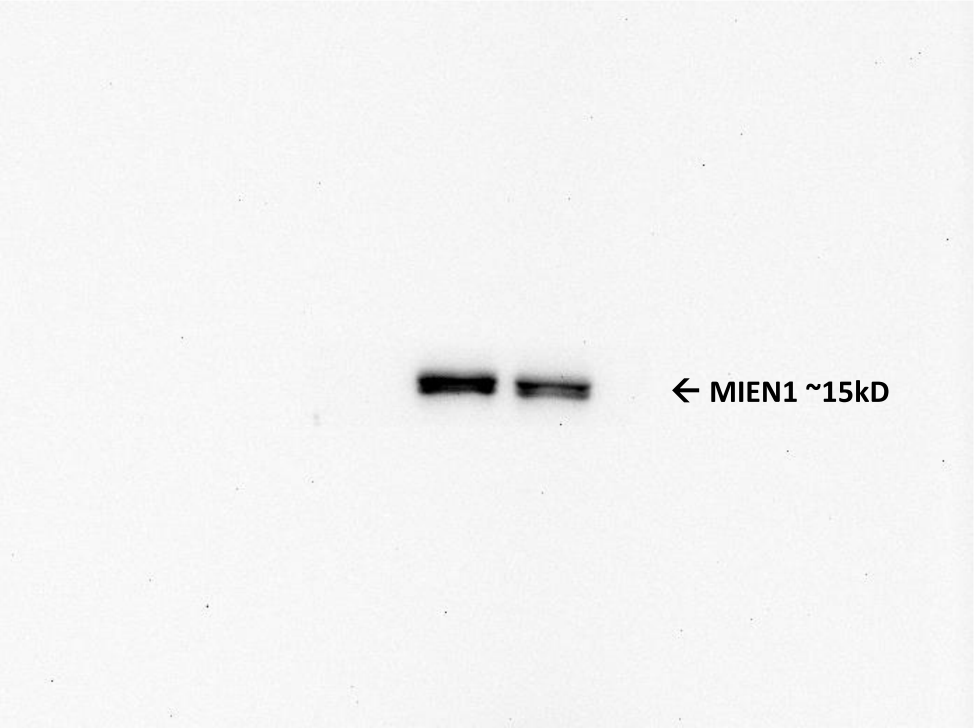

Actin (43kD)

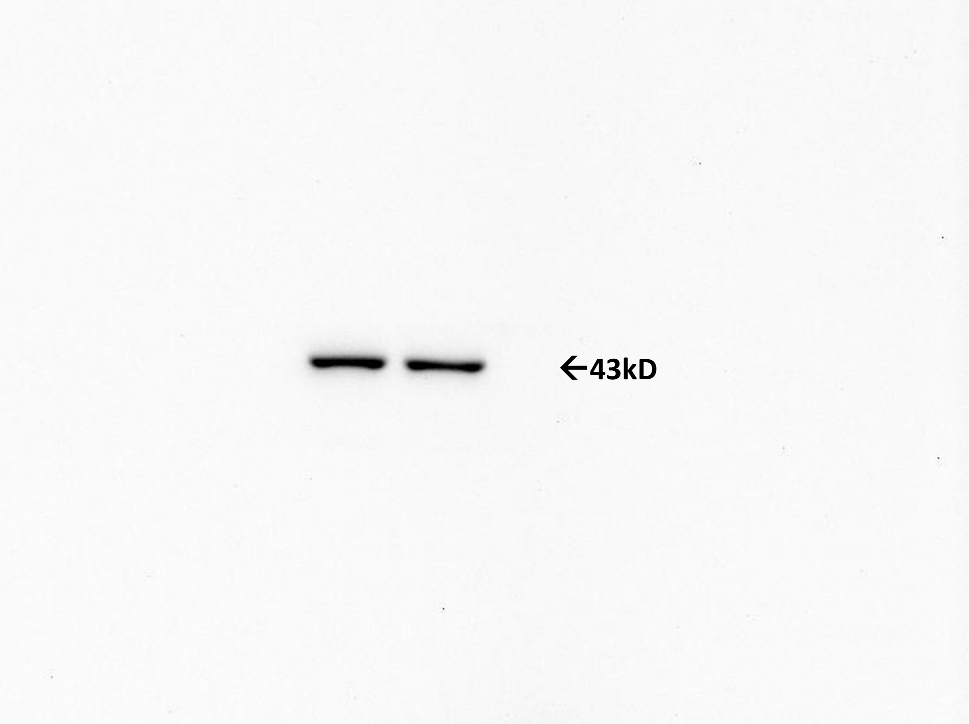

Figure 3G. PC-shCOL, PC-shMIEN1 (tumor)

MIEN1 (~15kD)

15kD (Marker)→

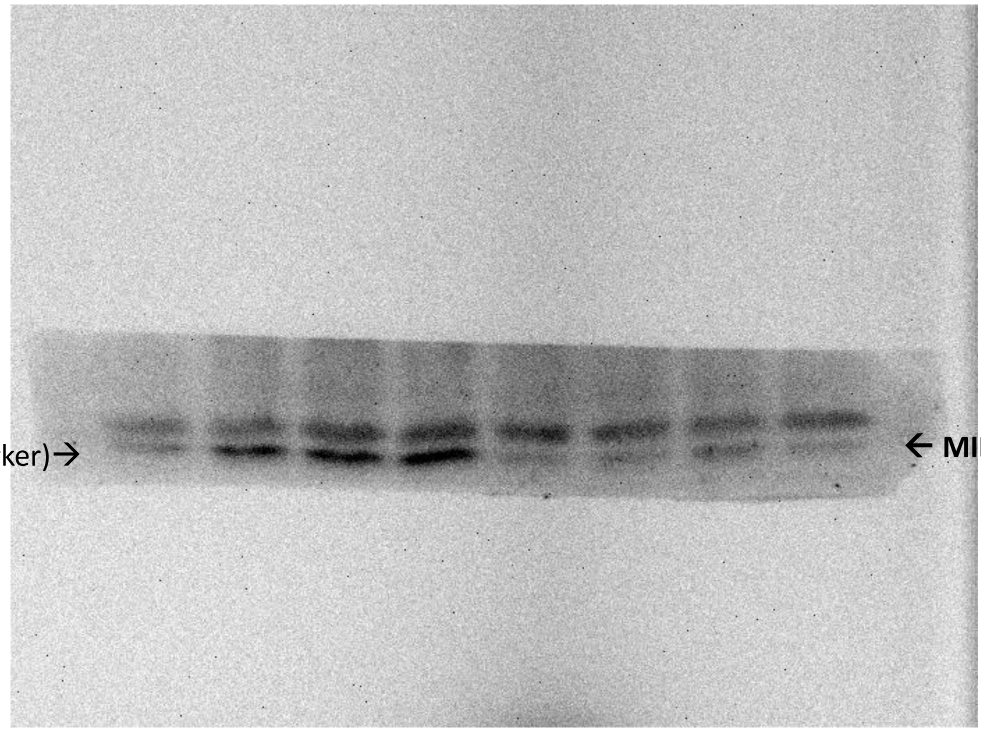

← MIEN1 ~15kD

Actin (43kD)

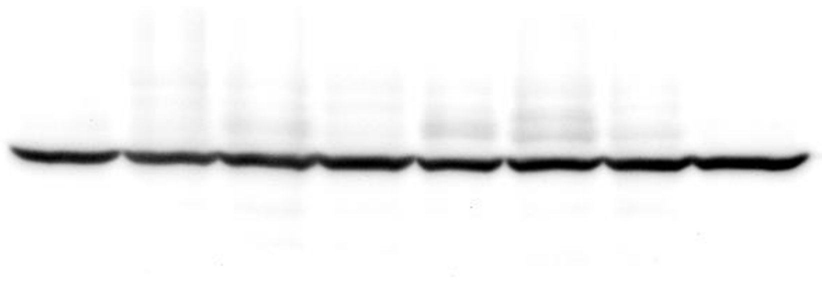

←43kD

Figure 5C. DU145-DNA, MIEN1-1,MIEN1-2

MIEN1 (~15kD)

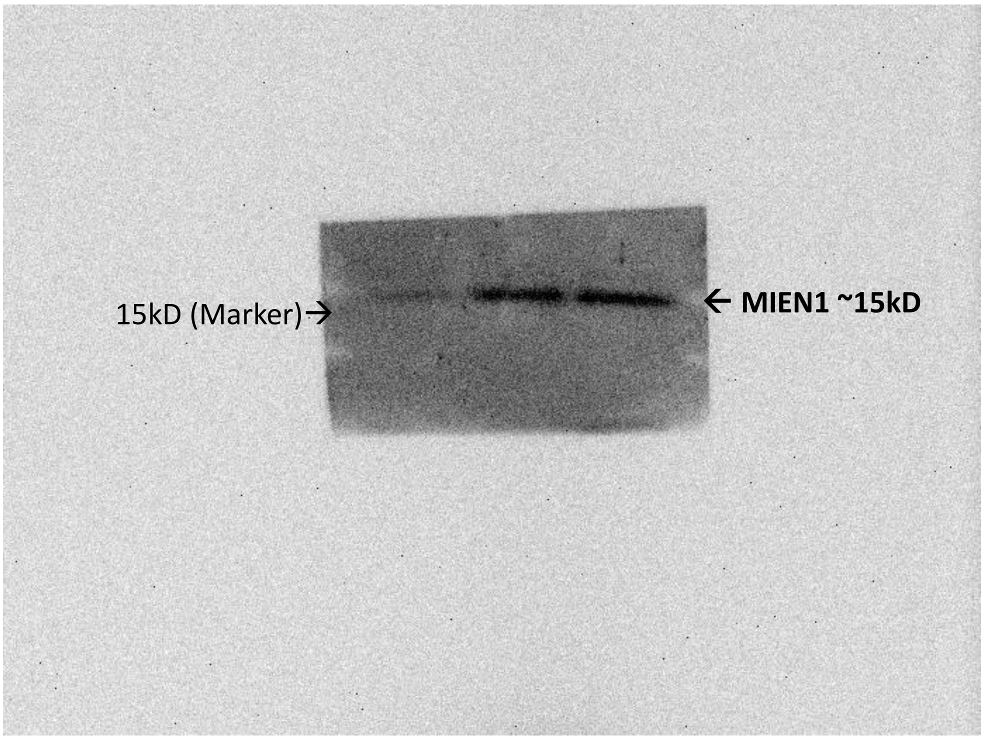

Actin (43kD)

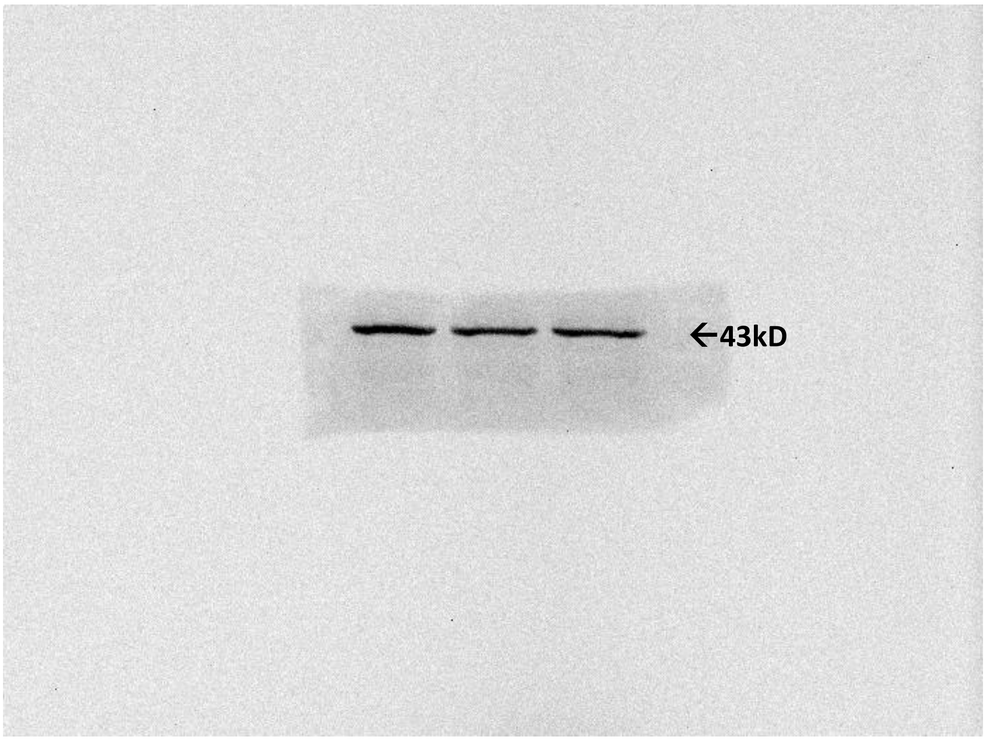

Figure 6H.   PC-shCOL, PC-shIL6

MIEN1

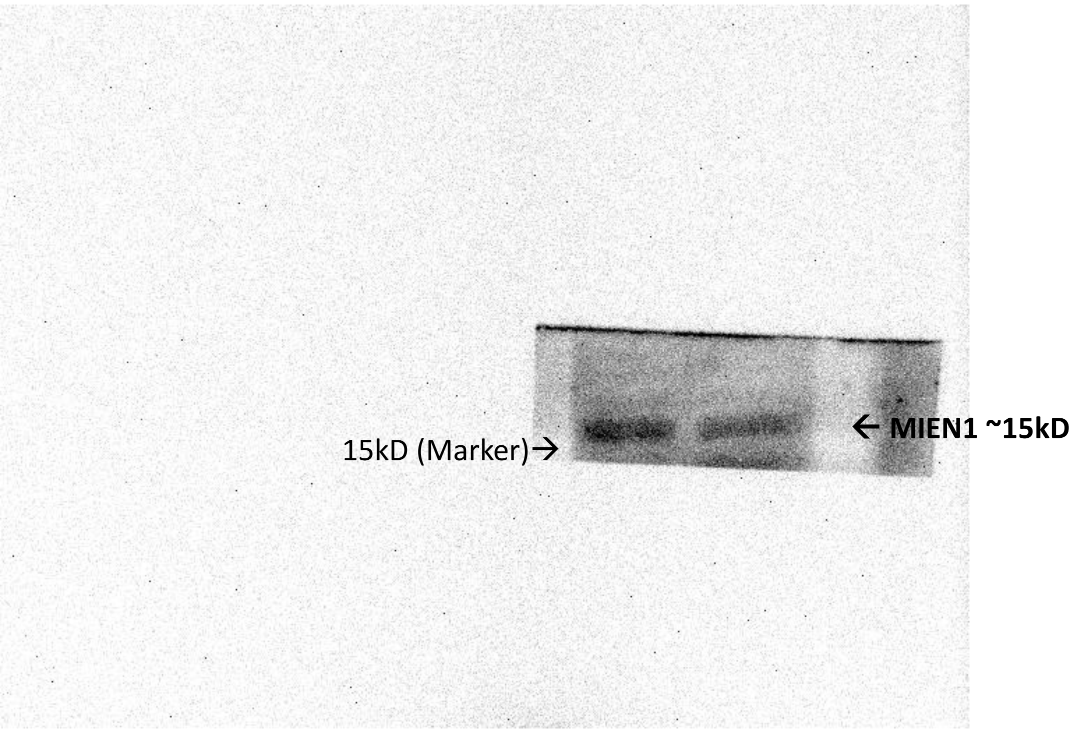

Actin (43kD)

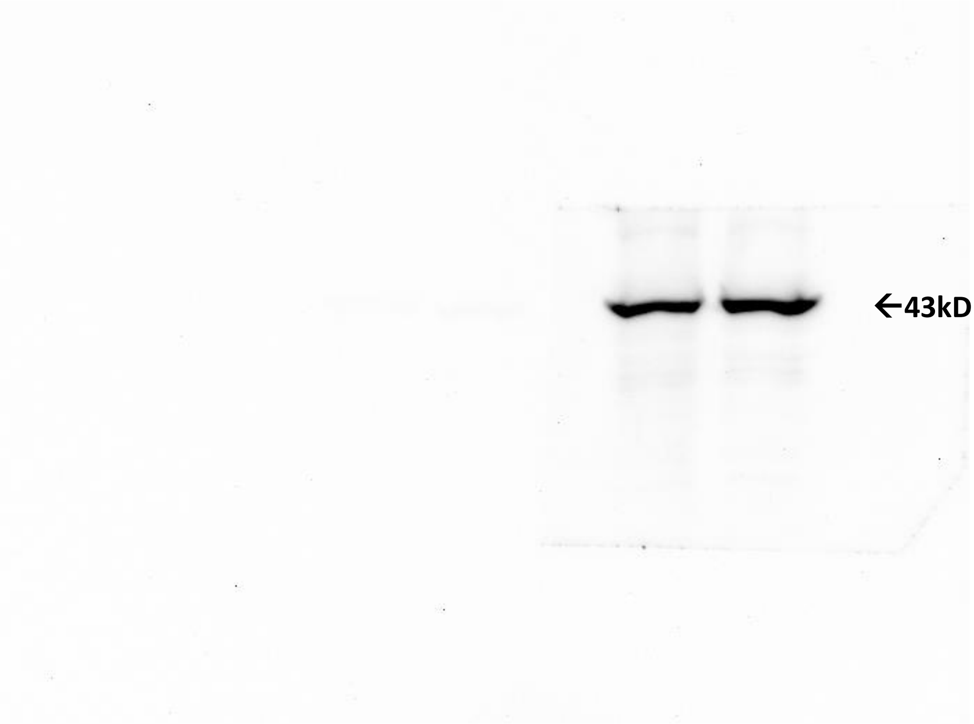

Figure 7A-1. PC-shCOL, PC-shMIEN1

NDRG1 (43kD)

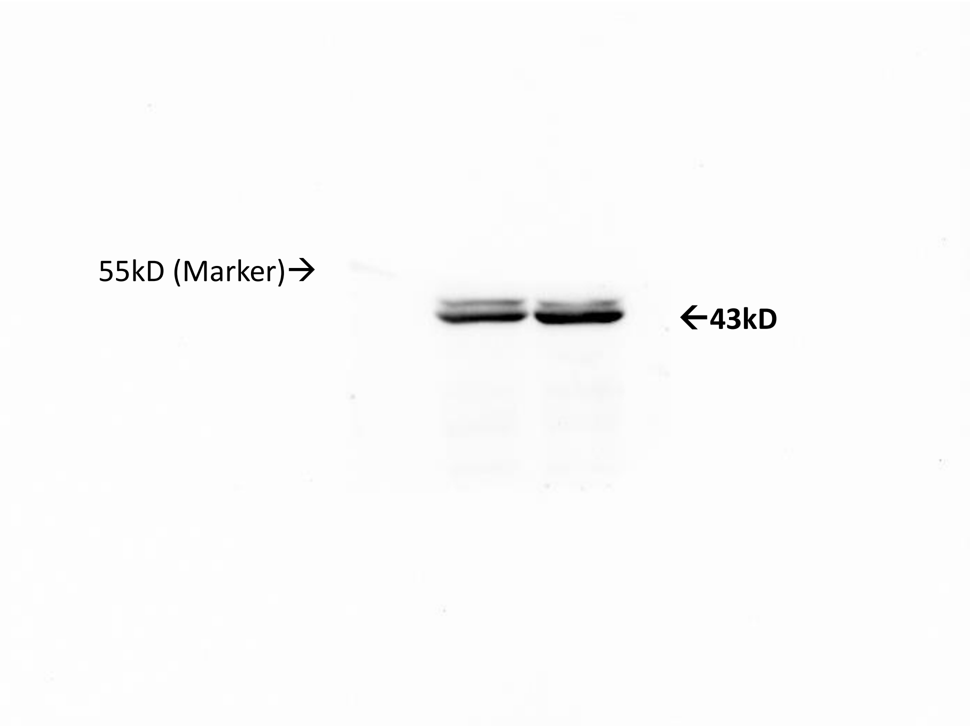

Actin (43kD)

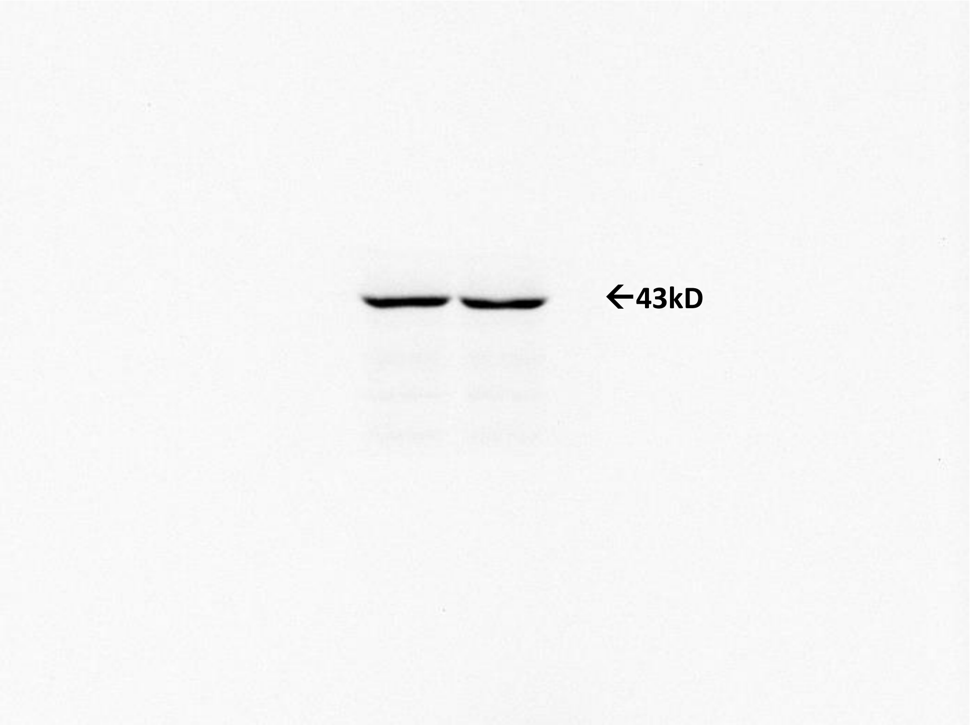

Figure 7A-2. PC-DNA, PC-MIEN1

NDRG1 (43kD)

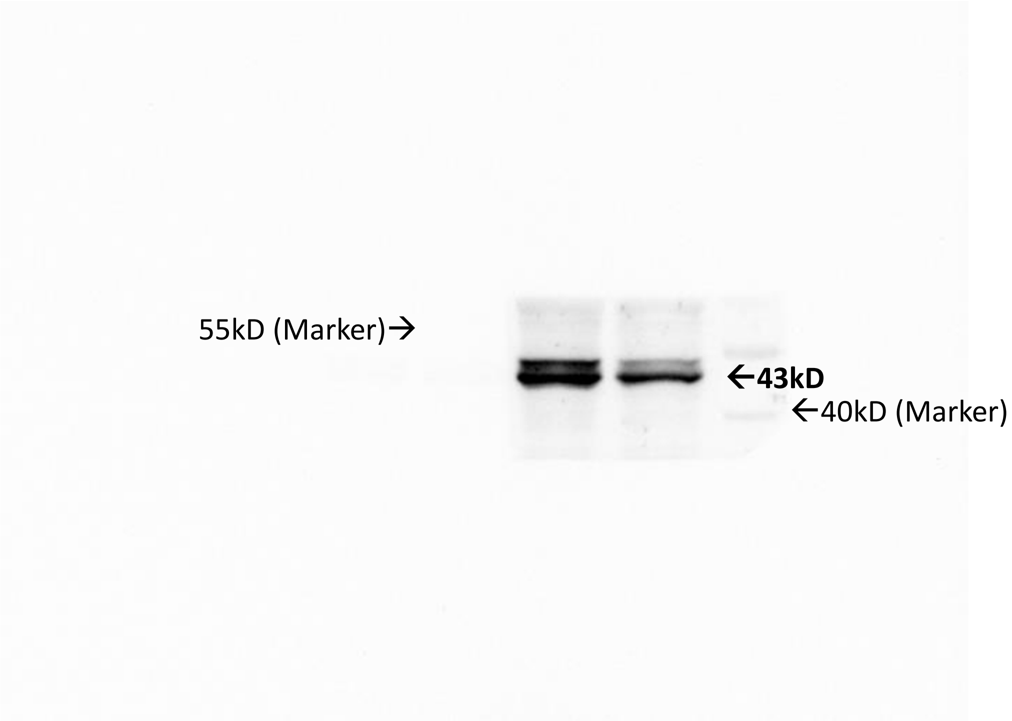

Actin (43kD)

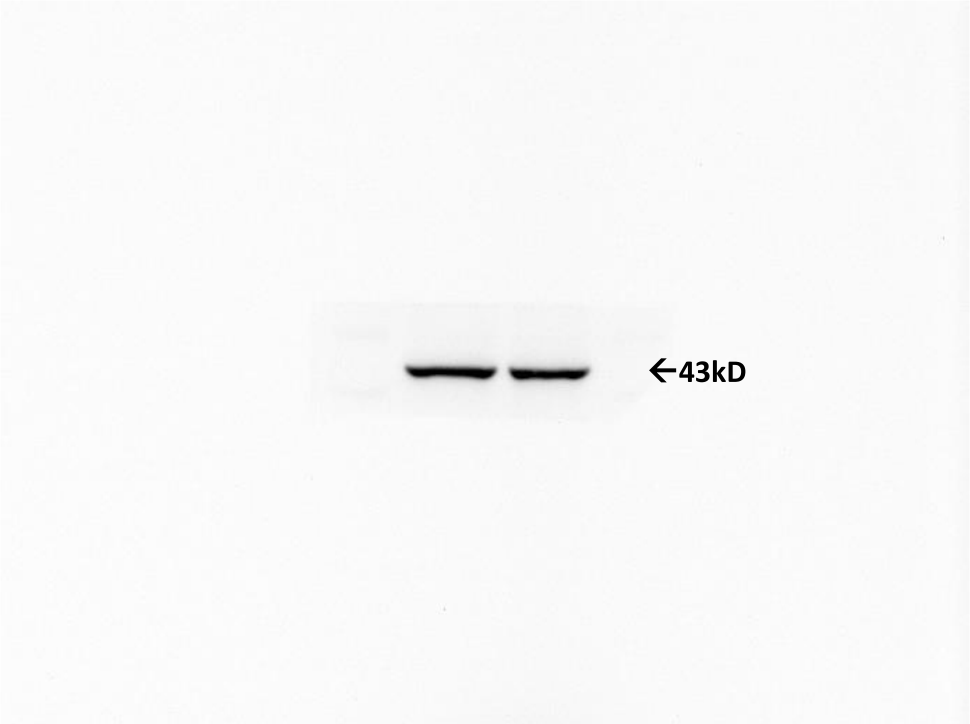

Figure 7B. LN-shCOL, LN-shMIEN1

NDRG1 (43kD)

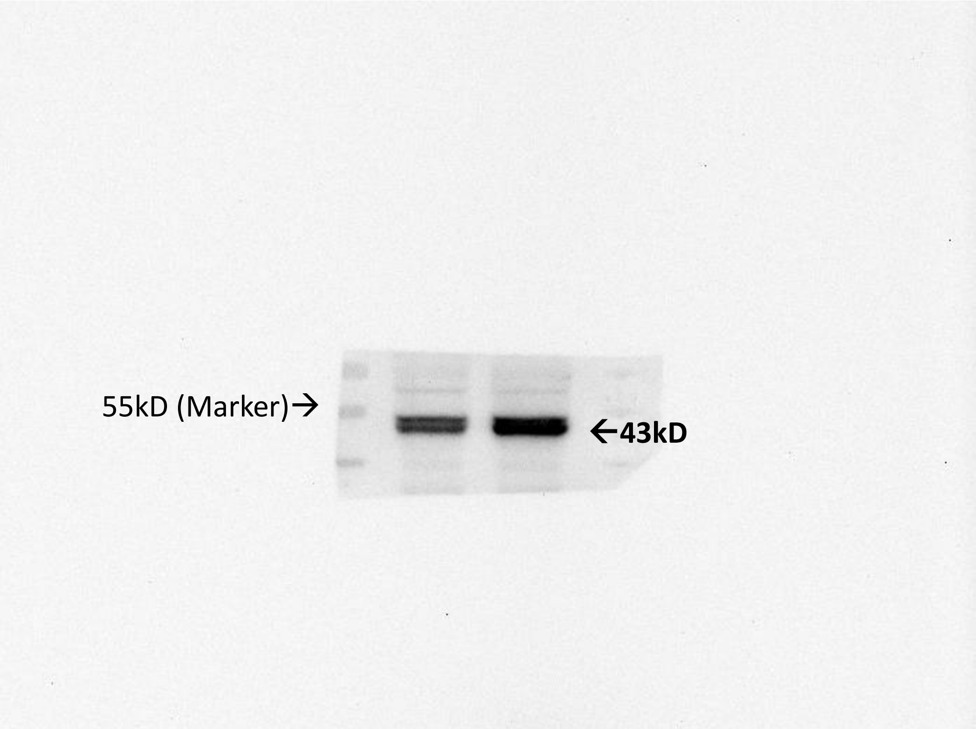

Actin (43kD)

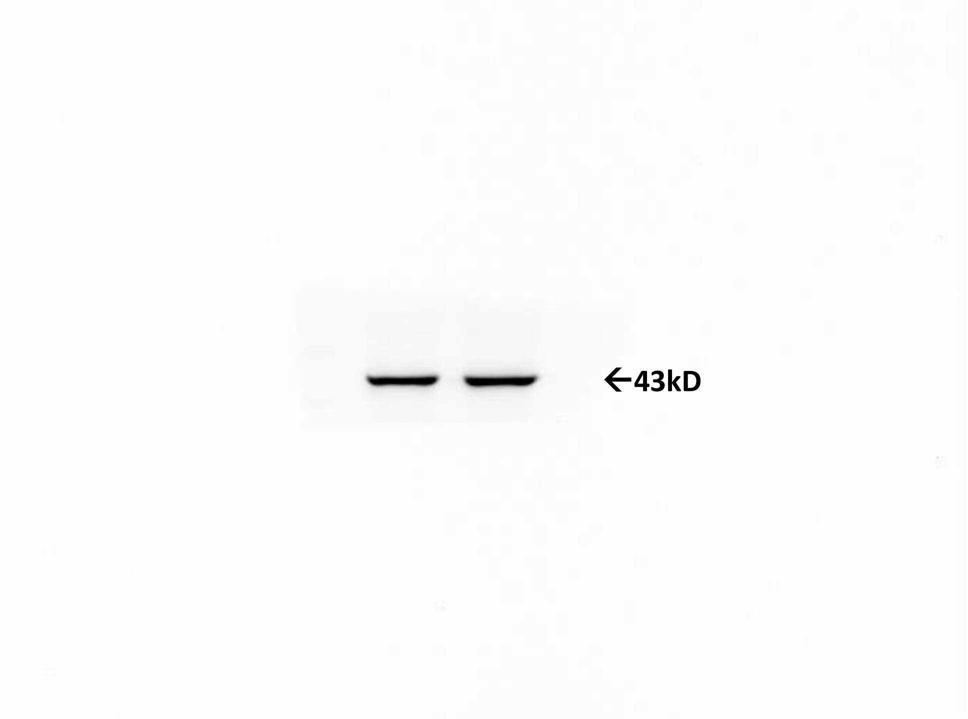

Figure 7D. PC-DNA, PC-MIEN1, PC-DNA+MK2206, PC-MIEN1+MK2206

NDRG1 (43kD)

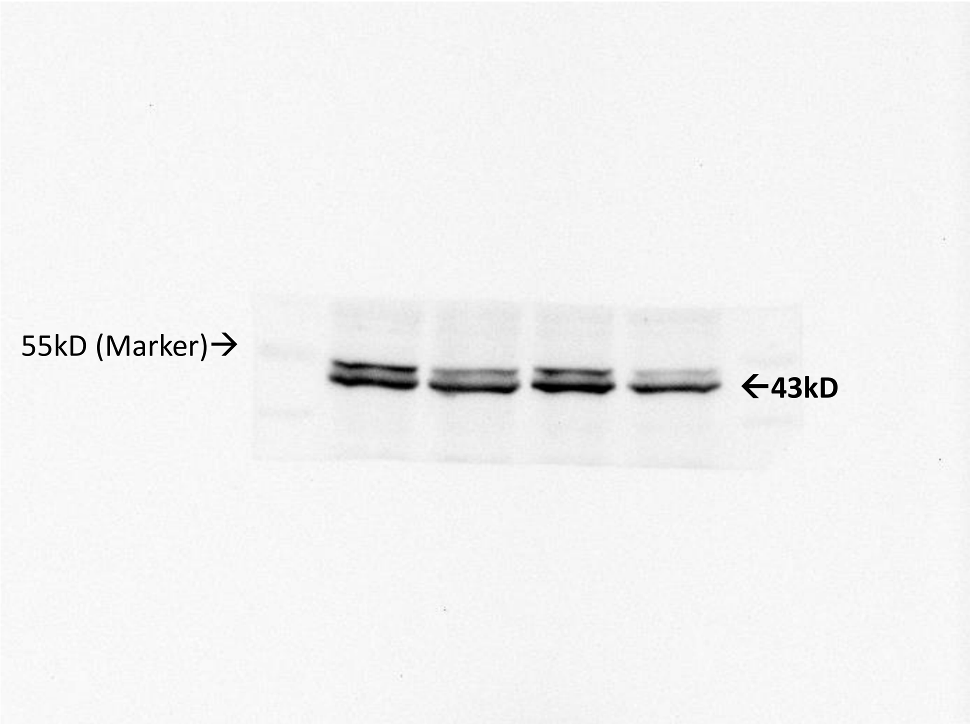

Actin (43kD)

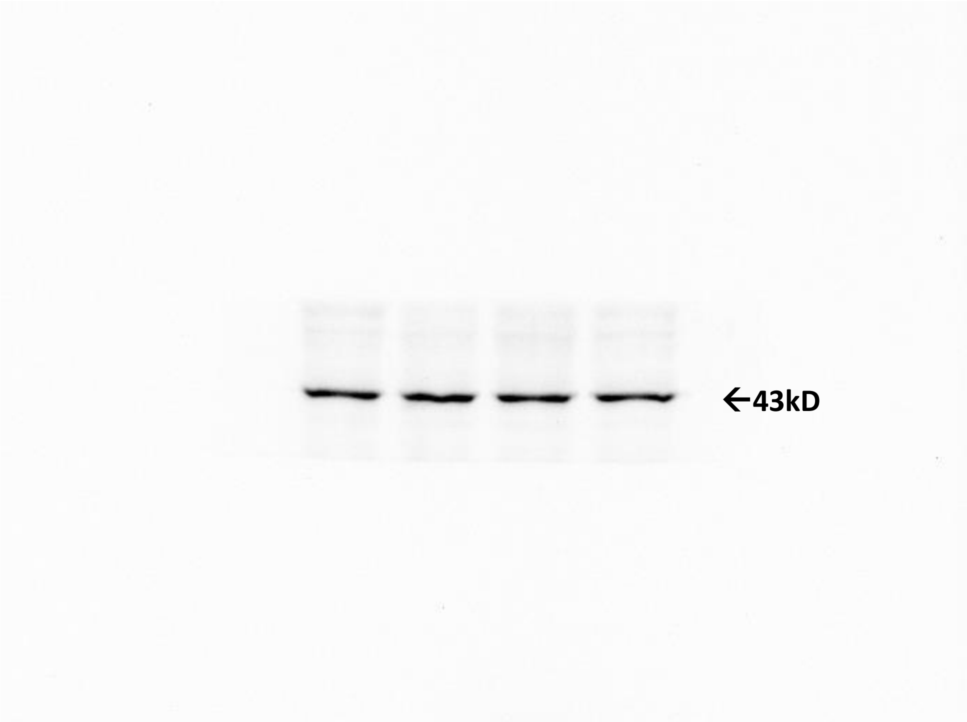

Figure S1. PC-DNA, PC-MIEN1

MIEN1 (~15kD)

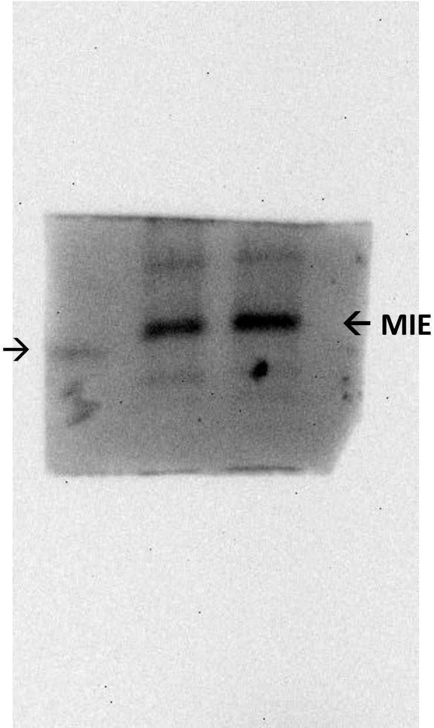

15kD (Marker)→

← MIEN1 ~15kD

Actin (43kD)

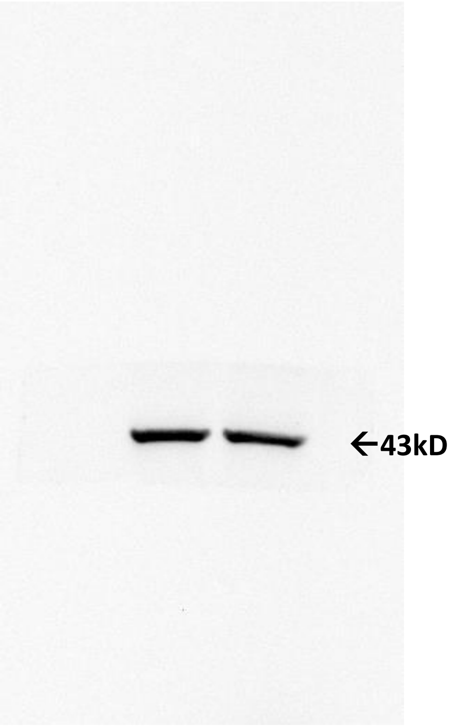

←43kD

E-cad (120-140kD)

180kD (Marker)→

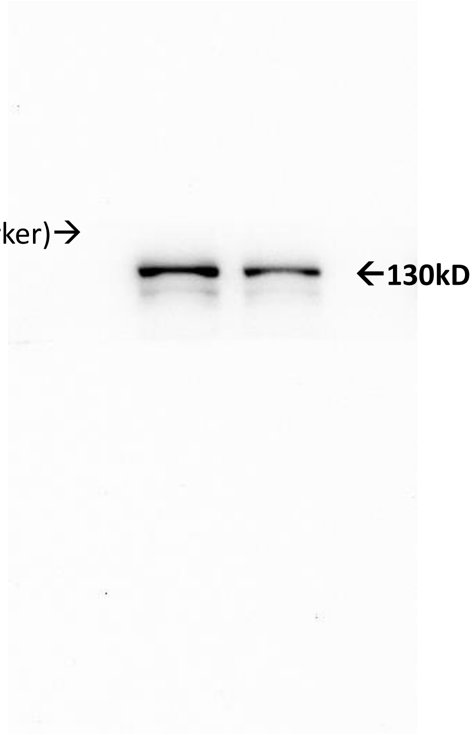

←130kD

N-cad (120-140kD)

180kD (Marker)→

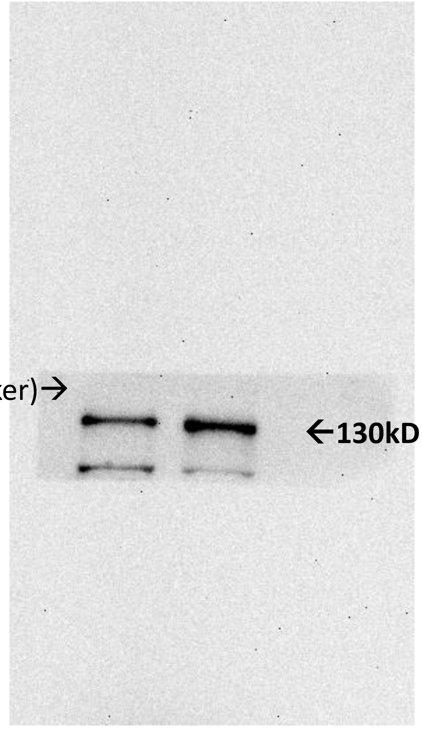

←130kD

Snail (29kD)

35kD (Marker)→

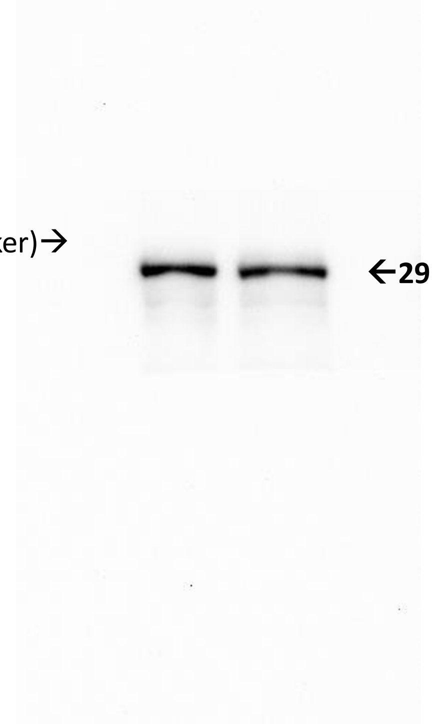

←**29kD**

Slug (29kD)

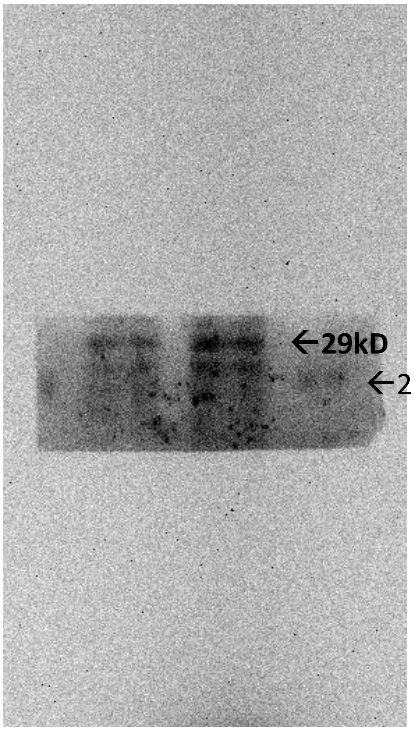

←**29kD**

←25kD (Marker)

Actin (43kD)

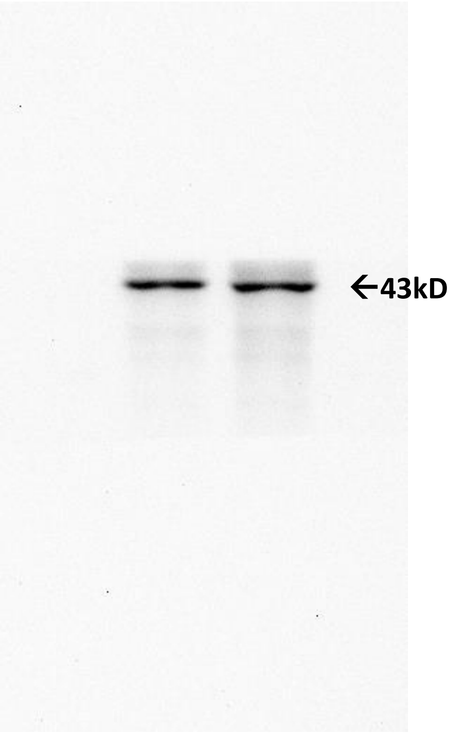

Supplement: Supplementary file 1 [file cancers-11-01486-s001.zip › cancers-600514-supplementary-revised/supplmemtary Figure 2.pdf]
